# Effects of the ‘10,000 Steps Duesseldorf' intervention promoting physical activity in community-dwelling adults: results of a nonrandomized controlled trial

**DOI:** 10.1186/s12966-025-01850-4

**Published:** 2025-12-03

**Authors:** Paula M. Matos Fialho, Elena Schmitz, Markus Vomhof, Andrea Icks, Alexander Lang, Oliver Kuss, Greet Cardon, Simone Weyers, Claudia R. Pischke

**Affiliations:** 1 Institute of Medical Sociology, Centre for Health and Society, Medical Faculty and University Hospital Duesseldorf, Heinrich Heine University Duesseldorf, Duesseldorf, Germany; 2 Institute for Health Services Research and Health Economics, Centre for Health and Society, Medical Faculty and University Hospital Duesseldorf, Heinrich Heine University Duesseldorf, Duesseldorf, Germany; 3 Institute for Health Services Research and Health Economics, German Diabetes Center, Leibniz Center for Diabetes Research at Heinrich Heine University Duesseldorf, Duesseldorf, Germany; 4 German Center for Diabetes Research, Partner Duesseldorf, Munich-Neuherberg, Germany; 5 Institute for Biometrics and Epidemiology, German Diabetes Center, Leibniz Center for Diabetes Research at Heinrich Heine University Duesseldorf, Duesseldorf, Germany; 6 Centre for Health and Society, Medical Faculty and University Hospital Duesseldorf, Heinrich Heine University Duesseldorf, Duesseldorf, Germany; 7 Department of Movement and Sports Sciences, Ghent University, Ghent, Belgium

**Keywords:** Physical activity, Population-based complex intervention, Replication study, Multi-level strategy

## Abstract

**Background:**

Despite approximately half of the German general population not meeting the physical activity (PA)-guidelines of the World Health Organization with further decreases noted during the pandemic, population-based intervention strategies to tackle this public health problem remain sparse. This study aimed to replicate the successfully delivered “10,000 Steps Ghent” intervention and research trial conducted in Belgium, two research questions were examined: (1) Do individuals located in the city that a complex PA intervention is implemented in (Duesseldorf) engage in more PA compared to individuals located in the control city (Wuppertal) after one year? (2) Is the proportion of those reaching 10,000 steps/day higher in the intervention than in the control city after the intervention?

**Methods:**

A nonrandomized controlled intervention trial was conducted among residents of two German cities. The 12-month intervention ‘10,000 Steps Duesseldorf’ was designed as a multicomponent intervention targeting different socioecological levels: at the intrapersonal level, a website allowed participants to self-monitor their steps; at the organizational level, workplaces and community groups were engaged through step count competitions; and at the community and policy levels, a city-wide media campaign to increase awareness regarding PA benefits was rolled out and street signage indicating walking routes were posted in different city district of Duesseldorf. To investigate intervention effects, PA was assessed via pedometers in two representative samples of adults aged 25–75 years from Duesseldorf (intervention) and Wuppertal (control). Measurements were taken at baseline (April 2021-March 2022) and again one year later (May–November 2023) and the same participants recorded their steps over a 7-day period at both time points. Baseline differences in socio-demographic and health-related variables between intervention and control were adjusted for in a propensity score model with matching weights.

**Results:**

627 adults completed baseline and 553 the follow-up assessment (60% female, 60% intervention across both timepoints and cities). The results of the propensity score analyses revealed that intervention participants walked an average of 462 steps per day more (95% confidence interval: -146 to 1070) than controls at follow-up. However, the proportion of residents reaching 10,000 steps/day was comparable between intervention and control (Duesseldorf: 26.4%, Wuppertal: 25.6%) after the intervention (odds ratio 1.04, 95% confidence interval: 0.41 to 2.66).

**Conclusions:**

Although the increase in step count detected in our study was promising, further intervention efforts and accompanying research are needed to meet the minimally relevant intervention effect of 1000 steps that was set in the original Ghent study.

**Trial registration:**

German Clinical Trials Register DRKS00024873 (Date of registration: April 21st, 2021; URL: https://drks.de/search/de/trial/DRKS00024873).

**Supplementary Information:**

The online version contains supplementary material available at 10.1186/s12966-025-01850-4.

## Background

One of the main risk factors for non-communicable disease mortality is a lack of regular physical activity (PA) [1]. In addition, long bouts of sitting are associated with physical impairments and linked to low levels of health-related quality of life [2]. The World Health Organisation (WHO) recommends that adults engage in 150 to 300 min of moderate-intensity PA per week and regularly interrupt prolonged sitting time by standing up or engaging in PA [1, 3]. Walking 7000 to 10,000 steps per day corresponds to approximately 150 min of moderate-intensity PA [4, 5]. Prior to the beginning of the COVID-19 pandemic, only 48% of German adults met the recommendations for PA formulated by the WHO at the time [6, 7]. The COVID-19 pandemic further exacerbated already insufficient PA-levels in the German general population preceding the pandemic. In Germany, lockdown measures, home-office regulations, and restrictions on sports facilities led to significant declines in PA and increases in sedentary behavior [8, 9]. National surveys reported reductions of up to 30% in moderate-to-vigorous PA, particularly among adults working from home, while leisure-time activities, such as walking and cycling, also decreased [10]. These declines are concerning given the established links between inactivity and both physical and mental health. Interventions that are simple, scalable, and community-based, such as step-count–focused strategies to boost PA (including outdoor activity), are therefore particularly well-suited to help counteract the long-term consequences of reduced PA during and after the pandemic. In adults, regular PA contributes to the prevention of noncommunicable diseases, such as cardiovascular diseases, cancer and diabetes and improves mental health and overall well-being [1].

The CARDIA-study found that in midlife walking 7000 or more steps per day was associated with a 50–70% lower risk of mortality in comparison with walking fewer than 7000 steps per day [11]. A meta-analysis of 15 longitudinal cohort studies supported this finding and showed an association of taking more steps per day and a progressively lower mortality. The risk is plateauing for younger adults (< 60 years) at 8000 to 10,000 steps per day and for older adults (≥ 60 years) at 6000 to 8000 daily steps [12]. Another recently published meta-analysis demonstrated a substantial inverse correlation between daily step count and both all-cause mortality and cardiovascular mortality, with higher step counts leading to greater benefits, particularly beyond the threshold of approximately 4000 steps per day for all-cause mortality and approximately 2000 steps for cardiovascular mortality [13].

Participation in population-based PA interventions that encourage the use of step counters has been shown to be associated with increases in steps/day [14, 15], particularly when a step goal of 10,000 steps/day is promoted. A review and meta-analysis of randomized controlled trials suggests increases in daily steps (mean difference (*MD)* = 554, 95%) (confidence interval (CI): 384–724) after 12 months of intervention (using devices to track PA) compared to control groups only receiving general health information which could be maintained for up to four years [16]. These findings were corroborated by another meta-analysis evaluating the effect of wearable activity trackers to promote PA in randomized controlled trials reporting an increase in daily steps (*MD* = 1078.53, 95% CI: 772.18 to 1384.87) [17].

In this context, multi-level, or complex, interventions play an important role. These approaches address not only individual behavior but also organizational, community, and structural factors, thereby targeting the broader determinants of health [18–20]. Studies examining their impact provide insights into real-world effectiveness, sustainability, and system-level impact that go beyond what can be demonstrated in randomized controlled trials [21, 22]. The “10,000 Steps Ghent” project exemplifies such an approach, illustrating how coordinated strategies across multiple levels can lead to meaningful and lasting improvements in PA behavior [23].

The complex community-based intervention ‘10,000 Steps Ghent’ was based on the social-ecological model [18, 23] and combined self-monitoring of PA with step counters (and documentation of steps via a website) with environmental intervention strategies (sign posts in public spaces with recommendations for walking routes, media campaign). It was evaluated between 2005 and 2006 in Ghent (intervention city) and Aalst (control city). In the Belgian study, an intervention effect was obtained with an average increase of 896 steps/day in Ghent (95% CI: 99 to 1192) and 8% of citizens in the intervention city (compared to the control city) reaching the 10,000 steps goal after one year of exposure to the complex population-based intervention [24]. The authors of this study concluded that even though effect sizes were relatively small (0.15–0.33), they were considerable for a whole-community intervention.

The aim of this replication study was to adapt ‘10,000 Steps Ghent’ to the German context and evaluate implementation and effectiveness of this community-based complex PA intervention among adults over a period of one year in two cities in North Rhine-Westphalia, namely Duesseldorf (intervention city) and Wuppertal (control city). Our hypothesis was that, similar to the original study, adults aged 25 to 75 years in the intervention city districts of Duesseldorf would show a greater increase in daily step counts compared to adults in the control city (Wuppertal) as a result of exposure to or uptake of the 1-year intervention. Furthermore, we assumed that the proportion of those achieving the WHO recommended PA target for adults, namely 10,000 steps per day [5], would be higher in the intervention city compared to the control city. The following research questions were examined in this study:

1. At baseline and after one year, do individuals in Duesseldorf (intervention city) differ in their average daily step count compared to individuals in Wuppertal (control city), and is the change in steps over time greater in the intervention city?
2. Does the proportion of adults achieving the recommended threshold of 10,000 steps per day increase more in the intervention than in the control city after one year of potential exposure to intervention activities?

## Methods

### Design and participants

This study had a non-randomized controlled trial design, as outlined in the published study protocol [25]. The intervention was implemented in selected city districts of Duesseldorf and residents of selected city districts in Wuppertal served as the control group. Participants were not individually randomized; instead, random samples of residents aged 25 to 75 years were drawn from the official population registers of both cities.

The intervention group consisted of residents of selected districts of Duesseldorf (Eller, Flingern Nord, Flingern Sued, Friedrichstadt, Gerresheim, Oberbilk and Wersten) and the control group of residents living in selected districts in Wuppertal (Elberfeld and Barmen). These two cities are located in the Western part of Germany in the state of North Rhine-Westphalia at a distance of approximately 30 kms (Duesseldorf: 655,000 inhabitants, Wuppertal: 365,000). Duesseldorf was selected as the intervention city because it is the location of our university, allowing for close collaboration with relevant city stakeholders and logistical feasibility. Local government authorities and community organizations were involved in the planning and implementation process through a stakeholder advisory board. Although the two cities are geographically close (~ 30 km), potential contamination was minimized as the intervention was implemented exclusively within Duesseldorf's administrative boundaries and communication channels, with no crossover activities or recruitment in Wuppertal.

Individuals were eligible for inclusion if they were between 25 and 75 years of age and resided in one of the selected intervention or control city districts. Districts were chosen based on publicly available socio-spatial deprivation indices to ensure comparability between the study sites. Individuals outside the defined age range or residing outside the selected city districts were excluded. Further details on the sampling strategy are provided in the published study protocol [25].

For each city, 2500 residents were randomly selected from the population registers of each city (500 for each age group of 25–35, 36–45, 46–55, 56–65, and 66–75 years) in the first wave. Equal proportions of men and women were included. An additional 2000 residents from each city were randomly selected in a second wave due to difficulties in recruiting and meeting the target during the COVID-19 pandemic. All potential participants were invited to participate in the study via mail. Further steps during recruitment are described in detail elsewhere [25]. All study participants were fully informed about the study and provided their informed consent. Ethics approval to conduct the study was obtained from the Ethics Committee of the Medical Faculty of the Heinrich-Heine-University Duesseldorf (reference number: 2021–1364; April 6, 2021). The intervention was implemented in Duesseldorf for one year, residents of the selected districts in the control city Wuppertal were not exposed to the intervention components described below.

### Data collection

Data collection was conducted at two time points: baseline (May 2021–March 2022) and 12-month follow-up (May–September 2023).

#### Interviews

After consent, participants completed a structured telephone-based interview (~ 1 h) with trained study nurses or student assistants. Interviews covered sociodemographic characteristics, health status, and PA, including the Global Physical Activity Questionnaire (GPAQ). All core questions were repeated at both time points. While telephone interviews were the primary mode, in rare cases questionnaires could be completed online.

#### Objective measurement of PA

After the interview, participants received a YAMAX EX210 pedometer by mail, worn during waking hours for seven consecutive days. Participants also completed a wear-time diary documenting daily step counts, wear periods, and reasons for non-wear time. Pedometers and diaries were returned using prepaid envelopes. To validate pedometer data, a subsample of 30% of participants also received an ActiGraph wGT3X-BT accelerometer, worn on the nondominant wrist for the same period. Accelerometer data were processed using ActiLife software and standard cut-offs for moderate-to-vigorous PA.

#### Confidentiality

All person-related data were pseudonymized by assigning unique study IDs. Identifiers were stored separately on secure, password-protected servers at the Heinrich-Heine-University Duesseldorf.

### Intervention

The "10,000 Steps Duesseldorf" intervention is a multi-level, or complex, intervention. It is based on socio-ecological models of health behavior, which emphasize that individual-level determinants are embedded in and influenced by broader interpersonal, organizational, community, and policy contexts. Unlike interventions that target only individual behavior change, multi-level approaches integrate strategies that address meso- and macro-level determinants simultaneously, such as organizational practices, community environments, and structural conditions.

Main messages, content, and components of the intervention “10,000 Steps Duesseldorf” were developed based on the original Belgian intervention “10,000 Steps Ghent” [24] which had been created based on the program "10,000 Steps Rockhampton" in Australia [26]. The participatory development process with city stakeholders in Duesseldorf is described elsewhere [27]. The main message of the intervention was “Every step counts”, the intervention goal was 10,000 steps/day. The intervention was implemented at different levels for one year (April 2022 to April 2023): intrapersonal level (including intrapersonal level components, such as website www.10000schritte-duesseldorf.de, online from April 2022 to March 2024 and printed materials to promote tracking of personal daily steps), and interpersonal components such as promotion of PA with family and friends, social comparisons via website, organisational level (step count competitions via website at workplaces and senior citizen centers), and community level (community events and local mass media campaign with a focus on regular PA, environmental street signs and walking circuits) [25]. A comparison of the socio-ecological intervention components between Germany, Belgium, and Australia is presented elsewhere [25].

### Intra and interpersonal level: Website ‘10,000 Steps Duesseldorf’

The content of the website was developed based on the content of the website of the former project [28] of the Flemish Institute of Healthy Living and Ghent University [24]. The website's content incorporates various behavior change techniques, including goal setting (behavior), highlighting the disparity between current behavior and the goal standard, self-monitoring of behavior, and social comparison [29]. Information about the benefits of regular PA and tips on how to integrate more steps into daily life were given on the website. As an essential part of the intervention, the voluntary use of step counters, wearable trackers or apps for self-monitoring as part of the behavior change strategy was promoted. The intervention website recommended various commercial trackers and apps across different price ranges, but these were not provided by the study team and not used for data collection. The website was developed jointly by the research team and a marketing company (crossactive gmbh). Current events focused on PA promotion planned by the research team (supplement 1) and those organized by the city of Duesseldorf were publicised on the website and information was frequently updated. In addition, intervention participants could create a personal profile to monitor daily steps, receive weekly overviews of steps, convert other activities to steps (e.g. swimming and bicycling) and compare step counts with others participants (interpersonal level) in step count challenges or competitions which were organized by the research team. Individuals could also create own step count competitions with family, friends, colleagues or other participants registered on the website.

The content of the website was, in part, tailored to the context of the pandemic. For example, residents were provided with information on how to re-establish PA routines while recovering from COVID-19 or on the important role of PA for immune functioning. Also, on the website, walks and walking routes with certain step counts were promoted that could be done even during restrictions which had to be followed during the pandemic.

### Organizational level: step count competitions and events for workplaces and senior citizen centers

Step count competitions for different organizations were implemented via the website (e.g., step count competition for workplaces in Duesseldorf organized by the Chamber of Commerce in November 2022, supplement 1). Based on the print media of the former project [24], flyers, booklets with step-count logs, stickers, posters were created [30]. The intervention material was available for download on the website and was distributed by student assistants at 3-month intervals in offices of general practitioners, physical therapists, pharmacies, newsstands and senior citizen centers located in the intervention city districts.

### Community and policy levels: community events, local mass media campaign, environmental street signs and walking circuits

During the intervention, different events to promote daily steps and step count competitions were held in the city of Duesseldorf at 2-month intervals (e.g., SkyRun climbing the steps of the Rhine-Tower) and in collaboration with the key city stakeholders (i.e., representatives of the Chamber of Commerce, the local professional soccer club Fortuna Duesseldorf, local city offices for Sports and Social Services). To promote the program and the PA events in Duesseldorf, regular posts were made on the program’s social media channels (Instagram and Facebook posts once per week). Also, articles appeared in local newspapers and radiobroadcasts on the program were made on local radio stations. Street signage was developed for the seven city districts indicating 20-min walking routes which were posted by the research team with permission of the City of Duesseldorf during the intervention period [30]. A QR-code to the website was displayed on the street signs. On the website, additional walking circuits were suggested via a Komoot^1^ account. For a detailed overview of activities planned and implemented during the one-year intervention period, see Supplement 1.

### Outcomes

The primary outcome was the number of steps taken per day. Participants were instructed to monitor their steps using the YAMAX EX210 pedometers which record daily step counts and have been validated in previous studies [31, 32]. Additionally, participants were asked to maintain a wear-time diary documenting daily steps, the duration the pedometer was worn, and reasons for any instances of non-wear.

Self-reported PA was assessed using the GPAQ administered during the structured telephone interview [33]. This questionnaire is widely employed and exhibits high levels of reliability and validity when compared to other PA questionnaires (International Physical Activity Questionnaire—Short Form and European Health Interview Survey—Physical Activity Questionnaire) [34]. Derived from the GPAQ, the assessment includes determining minutes spent on MVPA and various types of movements (for work, transport(ation), sports/leisure) [25]. The selection of this questionnaire was inspired by its use in the German National Cohort (NAKO) [35], a large German cohort.

### Covariates

The covariates age, gender, level of education, employment status, occupation time, distance to occupation, household income, migration background, marital status, partnership, type of housing were assessed with items from the NAKO core interview [35], the covariates changes in work time due to COVID-19, and changes in work due to COVID-19 came from the NAKO “Corona-questionnaire” [36]. The built environment was recorded with the ALPHA questionnaire [37] (self-reported environmental factors) and with items from the NAKO “Corona-questionnaire” [36] (changes in PA at work due to COVID-19, changes in PA in household due to COVID-19, changes in PA in leisure due to COVID-19, changes in PA in sports due to COVID-19, changes in PA in locomotion due to COVID-19, changes in PA in sitting due to COVID-19).

To assess participants’ health status, we used the following questionnaires: GEDA [7] (pre-existing comorbidities, smoking, intake of fruits and vegetables), modified version of the questionnaire of Chernyak et al. [38] (days in sick leave, hospital visits in the last 6 months), Alcohol Use Disorders Identification Test Short Version, translated based on the version from the PROMOTE I-study [39, 40], (alcohol intake), SOEP-Core-2018 (body mass index, self-reported state of health, and quality of life). The capability was assessed with ICECAP-A [41], and social support with items adapted based on Jackson et al., [42] and Fuchs [43]. The self-control score was assessed with reference to Sudeck and Pfeifer [44]. For more details see Matos Fialho et al. [25].

### Sample size

The minimal relevant intervention effect was set at 1000 steps. Based on the prior study by Cocker et al., [24] the standard deviation (SD) of the number of steps was ≤ 4500. This ensures 80% power for a two-sided t-test at the 5% significance level with a total of 638 participants (SAS 9.4, PROC POWER, TWOSAMPLEMEANS statement). To account for an anticipated dropout rate of 25%, the study included a total of 798 participants.

### Assignment method

As the size of the cities in North Rhine-Westphalia did not permit an assessment of all residents living in both cities, as was done in the original study [24], adjacent city districts were selected that encompass a similar number of residents as the cities included in the original study (for further detail, see Matos Fialho et al. [25]). Publicly available information on the levels of socio-spatial deprivation of the districts of Duesseldorf and Wuppertal was used [45, 46] during the selection process to ensure a composition of districts balanced by the proportions of residents living in them. The socio-spatial deprivation index is composed of indicators, such as proportion of residents receiving welfare benefits, living space per person, and proportion of residents with migration background in both cities. In Duesseldorf, the index can range from 1 (no deprivation) to 5 (high deprivation), and in Wuppertal, from 1 (no deprivation) to 4 (high deprivation) [46]. Corresponding to the heterogeneous population of the original study, the Duesseldorf districts of Flingern Nord, Flingern Sued, Oberbilk, Friedrichstadt, Gerresheim, Eller, and Wersten and the Wuppertal districts of Elberfeld and Barmen were chosen (Supplement 2). The residents of the selected districts of Wuppertal were assigned to the control group and the residents of the selected districts of Duesseldorf to the intervention group.

The smallest unit that is being used to analyse the intervention effect is the individual (i.e., residents of the intervention city Duesseldorf and residents of the control city Wuppertal who participated in the study). We selected the median number of daily steps across the seven recorded days as the primary outcome because it is less influenced by extreme values than the mean, and, thus, more accurately reflects typical daily activity [11, 47–51].

### Statistical methods

Continuous variables are described as means and standard deviations and categorical variables as absolute and relative frequencies. The primary outcome was calculated as the difference in the median daily steps over the seven reported days with corresponding 95% confidence intervals (95% CI) for the participants in the intervention and control cities at follow-up. In addition, as the secondary outcome, we computed the odds ratio (with 95% CI) of achieving more than 10,000 daily steps in the intervention group. To account for the non-randomized assignment of the intervention, we used the propensity score (PS) method for the primary analysis of the intervention effect. Specifically, we used the matching weights method [52] with matching weights calculated from a PS model including 65 baseline covariates to comprehensively reduce confounding (Supplement 3). To assess balancing of covariates before and after applying matching weights, we calculated standardized z-differences [53] using the *weightedZdiff* package in R [54]. Smaller z-differences are indicating a better balance between groups. In the weighted population, balance improved for 60 of the 65 baseline covariates while the remaining five covariates already had z-differences very close to zero, thus were already well balanced. Missing values of covariates were replaced by imputation. Due to the small percentage of missing values (average/maximum proportion of missing values per covariate 0.5%/7%) only a single imputation step was performed.

In sensitivity analysis, the intervention effect on the primary outcome was investigated using single imputation also for the median number of steps at follow-up for participants that had baseline data but missing values for the primary outcome at follow-up. All data were analysed with SAS (version 9.4) and z-differences were calculated in R 4.0.5. This manuscript was written following the TIDieR checklist (Supplement 4).

## Results

### Participant flow

**Fig. 1:**
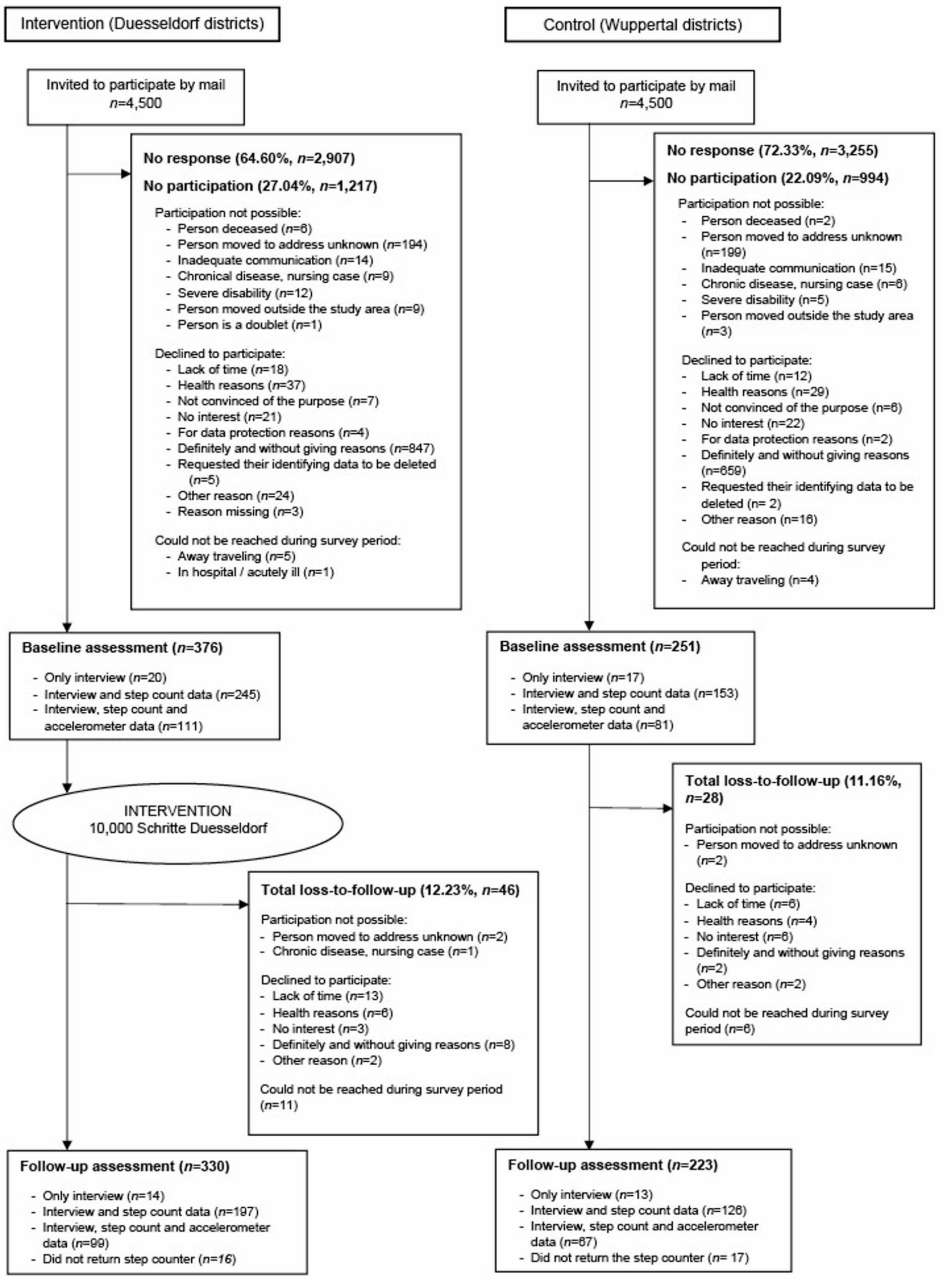
Flow chart

Participant flow is shown in Fig. 1. Of 9000 invited residents (*n* = 4500, *n* = 2500 in each city in wave 1 and *n* = 2000 in wave 2, also see detailed description in the study protocol [25]), the non-response proportion was 65% of the sample in the intervention and 72% in the control city. Of residents providing a telephone number to determine study eligibility, 27% were deemed ineligible or declined to participate in the intervention or could not be reached during the survey period, this was the case for 22% in the control condition. The baseline data were collected in May 2021 to March 2022 and follow-up data in May to December 2023. 627 participants (Duesseldorf: 60%, *n* = 376; Wuppertal: 40%, *n* = 251) completed the baseline interview. Of the 627 participants, 356 provided pedometer-based step count data in Duesseldorf and 234 in Wuppertal. The overall response proportion in the study was 7% (Duesseldorf: 9%; Wuppertal: 6%). The total loss-to-follow-up at one year was 12% (n = 74). Five-hundred and fifty-three participants completed the interviews at one-year follow-up (Duesseldorf: 60%, *n* = 330; Wuppertal: 40%, *n* = 223). The step count data of 296 participants in Duesseldorf and 193 in Wuppertal were obtained at one year.

### Baseline data and equivalence

Table 1 displays the demographic baseline characteristics of participants included in the intervention and control group before and after application of the matching weights including z-differences. The average age of participants was 55.3 years (SD = 13.8) in Duesseldorf and 54.6 years (SD = 13.4) in Wuppertal. In terms of gender distribution, 62% of the participants in Duesseldorf and 57% in Wuppertal were women. Overall, 50% of the sample held a university degree, while 32% of participants in Duesseldorf and 36% in Wuppertal reported having a migration background. The complete list of all 65 baseline covariates in the PS model is given in the Supplement 3. In the weighted groups, covariate balance was improved for the majority of covariates (91% had a better covariate balance) compared to the study population without applying matching weights.

**Table 1:** Characteristics of participants in the intervention (Duesseldorf, *n* = 376) and control group (Wuppertal, *n* = 251) before and after applying matching weights

Demographics	Duesseldorf baseline (*n* = 376)	Wuppertal baseline (*n* = 251)	z-difference	Duesseldorf baseline with matching weights (*n* = 376)	Wuppertal baseline with matching weights (*n* = 251)	z-difference
Age (years)						
Years (mean ± SD)	55.3 ± 13.8	54.6 ± 13.4	−0.67	55.6 ± 5.4	55.7 ± 6.2	0.02
Gender (%)						
Women	62%	57%	−1.26	61%	61%	−0.05
Level of education (%)						
College or university degree	53%	56%	0.79*	54%	54%	0.06*
Employment status (%)						
Employed	60%	71%	−2.79*	66%	64%	0.31*
Household income (Euro)						
Euro (mean ± SD)	2895 ± 1145	2660 ± 1518	−1.07	2683 ± 612	2681 ± 773	−0.01
Migration background (%)						
Yes	32%	35%	−0.83*	35%	38%	−0.43*
Body mass index (kg/m^2^)						
Kg/m^2^ (mean ± SD)	25.8 ± 4.9	25.9 ± 4.6	0.66	26.1 ± 1.7	26.1 ± 2.3	−0.21
Smoking (%)						
Daily	10%	15%	−1.72	13%	13%	0.12
Sometimes	4%	3%		4%	3%	
Former	36%	38%		34%	34%	
Never	50%	44%		50%	51%	
Self-reported state of health (%)						
Excellent	5%	5%	0.38	4%	4%	0.26
Very good	27%	27%		25%	26%	
Good	53%	51%		53%	51%	
Poor	12%	16%		16%	17%	
Weak	3%	1%		2%	2%	
Self-reported PA (MET-min/week)						
Work-related intensive PA	635 ± 1983	800 ± 2561	0.20	716 ± 765	747 ± 1071	−0.11
Work-related moderate PA	318 ± 991	400 ± 1280	0.20	358 ± 382	373 ± 535	−0.11
Locomotion by foot or bicycle	856 ± 973	822 ± 1012	−1.46	837 ± 356	868 ± 473	0.22
Leisure time intensive PA	643 ± 1004	935 ± 1587	1.83	695 ± 405	733 ± 601	−0.10
Leisure time moderate PA	701 ± 1246	824 ± 1194	2.09	803 ± 612	834 ± 510	1.02
Sedentary behavior (min/day)	409 ± 204	419 ± 198	0.72	405 ± 82	409 ± 85	0.41
Pedometer-determined PA (steps/day)	7989 ± 3358	8022 ± 3640	−0.28	8129 ± 1455	8163 ± 1842	−0.13

Continuous variables are described as means and standard deviations and categorical variables as relative frequencies

*MET* metabolic equivalent of task, *PA* physical activity

^*^based on the complete variable including all categories, simplified for presentation of characteristics

### Outcomes and estimation

**Fig. 2:**
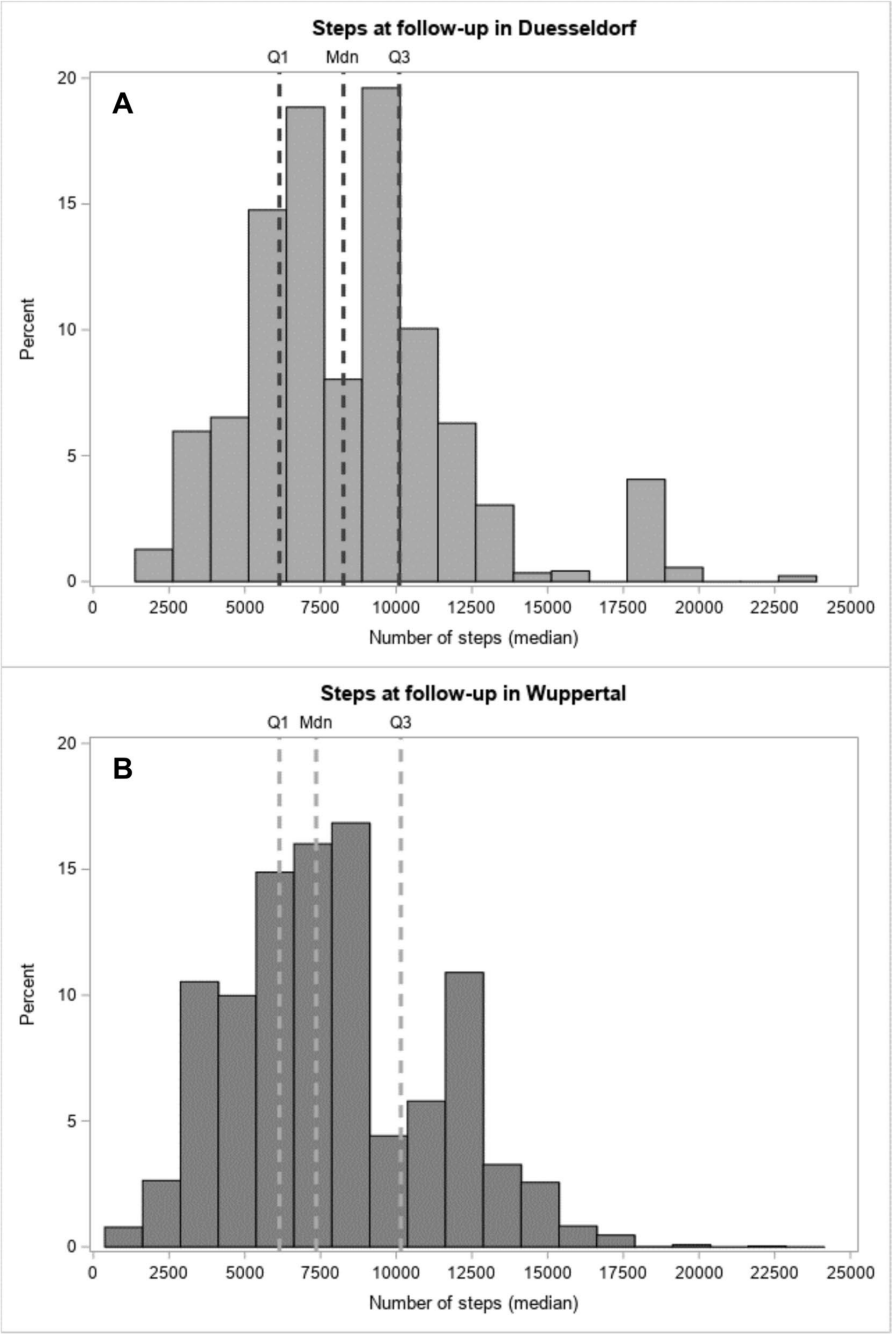
Number of steps (median over the reported seven days) in the intervention (A—Duesseldorf) and control city (B—Wuppertal) at follow-up assessed with a pedometer. Numbers are weighted by the respective matching weights.Mdn, median; Q1, first quartile; Q3, third quartile

The median numbers of daily steps at follow-up after the intervention in Duesseldorf and Wuppertal are shown in Fig. 2. After PS weighting, participants in the intervention groups walked 462 steps/day more (95% CI: −146 to 1070) at follow-up as compared to the control group. The proportion of participants reaching ≥ 10,000 steps/day at follow-up was comparable between both cities (Duesseldorf: 26.4%, Wuppertal: 25.6%). The corresponding odds ratio was 1.04 (95% CI: 0.41, 2.66), indicating that participants in Duesseldorf had similar odds of achieving ≥ 10,000 steps/day when compared with those in Wuppertal (reference group). In the sensitivity analysis, in which data on steps at follow-up were imputed for all baseline participants with missing data on the primary outcome, the intervention effect was comparable (636 steps/day, 95% CI: 77 to 1194).

## Discussion

The main result of our non-randomized controlled intervention study was that after one year, participants from Duesseldorf walked 462 steps/day more (95% CI: −146 to 1070) at follow-up than participants in the control city of Wuppertal. Hence, the absolute number of steps taken per week increased more for individuals residing in the intervention than in the control city. However, our point estimate is smaller than that reported in the original Ghent finding an average daily step increase of 896 (95% CI: 599 to 1192) in the intervention community after one year [24]. Further, contrary to the original study, we did not find a higher proportion of participants reaching the “10,000 steps" goal in the intervention group after 1 year of exposure to intervention messages and activities compared to control. In fact, the proportion of study participants with $\ge$ 10,000 steps/day at follow-up was comparable between both cities (Duesseldorf: 26.4%, Wuppertal: 25.6%), the odds ratio was 1.04 (95% CI: 0.41 to 2.66, reference: Wuppertal).

Although the intervention group showed a higher mean step count than the control group, the difference was modest. Therefore, while the results are promising and point in the expected direction, they should be interpreted with caution regarding statistical significance. Nevertheless, we emphasize that the intervention effect was quantified through its point estimate and confidence interval, an approach nearly universally (e.g., in essentially all reporting guidelines) recommended in biomedical research as a more informative alternative to the mere dichotomized result of a statistical test [55].

When interpreting these results, we ought to consider that the socio-demographic composition of selected city districts in the intervention and control groups corresponds to the sample of the original Ghent study. Our samples were randomly selected from the population registers and well balanced in age and gender. In a second recruitment wave during the pandemic, we were able to reach the required sample size and, with 12%, the loss-to-follow-up was acceptable. The primary limitation of the study, however, was that it was conducted during the COVID-19 pandemic. Especially during the time of the baseline assessment (April 2021 until March 2022) several restrictions, such as quarantine and social distancing, were still ongoing in Germany. The pandemic led to a selection bias in our sample where physically active participants were overrepresented in our study. Starting with high baseline values did not leave much room for improvement at follow up.

While the 10,000-steps-per-day target is a simple and widely recognised way to promote PA and aligns well with our intervention slogan “Every step counts”, it may not have been equally meaningful to all participants. Those who were already close to or had surpassed 10,000 steps at baseline were unlikely to have had much incentive or scope to increase this figure further. In contrast, participants who started from lower levels of baseline activity had greater potential to benefit. The variability in baseline steps in our sample (Table 1) underscores this point. This suggests that having only one absolute limit may have reduced the ability to respond effectively for some subgroups. Alternative strategies, such as encouraging incremental increases relative to baseline activity (e.g., an additional 2000 steps per day), have been suggested as more motivating [5, 15]. In our study, baseline step counts averaged at approximately 8000 steps compared to 6600 (intervention city) and 6961 (control city) in the original Ghent study leaving less room for improvement. High baseline PA-levels of our sample were probably due to more active individuals deciding to participate in the study during the pandemic.

Regarding our main outcome, the use of pedometers should have led to precise measurements. However, we cannot rule out social desirability in the documentation of daily steps in this already relatively physically active population. It should be noted, however, that results on PA measured with accelerometers from the NAKO suggest comparatively high levels of PA measured via accelerometers. Here, women and men aged 20 + years reported engaging in moderate to intense PA for 75 min and 73 min per day, respectively [35] (corresponding to ~ 5000 steps/day compared to ~ 8000 ± 1600 steps/day in our study, analysis of accelerometer data currently underway). This supports the notion that individuals with an already active lifestyle tend to self-select into studies on health, such as NAKO or our study.

A strength of our study was that using the propensity score method with 65 baseline covariates led to a nearly perfectly balanced study sample with achieving balance for the most clinically relevant and known confounders. In addition, applying matching weights allowed for estimating the intervention effect with a minimal variance [52]. The effectiveness of the complex intervention on daily steps is plausible. We stuck closely to the original intervention which was based on the social-ecological model [18] and adapted all intervention levels (intrapersonal, interpersonal, organisational, community, policy) [25] to the local context of Duesseldorf. This adaptation process was supported by a network of local stakeholders [27]. However, the COVID-19 pandemic influenced people’s PA in different ways. Most studies indicated significant decreases in mobility, walking, and PA, but few studies also reported increased use of parks/trails and more recreational activity among certain populations [56]. We cannot disentangle the impact of the intervention and the pandemic on daily steps. However, it can be argued that residents in both cities were similarly affected by the pandemic.

One major ingredient of our Intervention was the intervention message “Every step counts” and the recommendation to track steps and to use wearable activity trackers or apps to self-monitor daily steps. Specifically, on the website, recommendations on trackers and apps spanning a certain price range were made. Interestingly, the point estimate reported for our study is broadly consistent with a pre-pandemic meta-analysis of 28 trials [14] examining wearable activity tracker–based interventions, which reported increases in daily steps compared with control groups. However, analyses split up by length of intervention exposure (≤ 12 weeks, number of studies = 23 vs. > 12 weeks, number of studies = 9) suggest increases of steps approximately twice as high as those found in our study up to 12 weeks post-intervention (MD = 1358, 95% CI: 950–1766). Beyond 12 weeks, step count increases were comparable to our study. Intervention participants, on average, walked 522 steps more than at baseline (MD = 522, 95% CI: 258–785). However, interventions only consisted of wearable activity trackers, no other intervention components comparable to those implemented in our study were evaluated which somewhat limits the comparability of effects.

Wahlich et al. (2020) conducted a review of trials involving adults with objective PA measures and a follow-up period of at least 12 months. Nine studies were included in the review, selected from an initial set of 17,233 records. The interventions ranged from individual walking programs to group-based sessions and school volunteering with follow-ups ranging from 12 months to four years. Pooled analyses of four studies showed a modest increase in PA at 12 months for intervention participants compared to control participants (MD: 554 steps per day; 95% CI: 384–724) [16], demonstrating that structured interventions can produce sustained improvements in activity levels. Another review of long-term, multimodal PA interventions reported an increase of approximately 2197 steps per day after 12 months [57]. However, these studies mainly involved individual-level counselling and did not integrate community, media or environmental strategies. Our study builds on this body of research, evaluating a community-based, multi-component intervention, the background being that broader, real-world approaches may deliver greater and more lasting improvements in PA than simpler, individual-focused programs. With a + 494 steps/day increase, the finding of our study was comparable to the step count increases noted after participation in tracker-based interventions. However, more research is needed to evaluate the long-term use and impact of our complex intervention in the city of Duesseldorf.

Due to the support of the local stakeholder network in Duesseldorf, we could implement all intervention components at the intended levels and as planned during the participatory phase preceding the study (see Matos Fialho et al.) [25]. Delays in activities at the start of the intervention due to the pandemic and associated restrictions, such as travel bans for involved stakeholders and participants, could be made up for by extending the intervention period so that the intended 1 year of intervention activities was still reached. Activities at the organizational level (i.e. at workplaces and senior citizen centers in intervention districts) were disproportionately affected by restrictions, such as closures. In contrast, other intervention levels, such as website use, roll out of the media campaign, and posts of environmental street signs were not affected. The sample bias led to a limited generalizability of the trial findings. In addition, only short-term effects were examined in this study as compared to the original study that rolled out the intervention to the entire state of Flanders after ten years [58].

## Conclusions

Based on the rather small increases in step counts after one year found in residents of the intervention (vs. control) city districts in our study in comparison to the original Ghent study, we argue in favor of future studies to be conducted in the German urban context that do not take place under pandemic restrictions and that cover longer periods of time as was the case in Flanders [58]. This future research effort would be worthwhile as previous evidence suggests that increases of + 494 steps/day from both individual and group-based interventions can be maintained for up to four years [16]. Thus, efforts are currently underway to permanently establish intervention components of the complex intervention examined in this study at the Public Health Office of Duesseldorf.

## Supplementary Information

Supplementary Material 1. Intervention components planned and implemented

Supplementary Material 2. City districts chosen for the study in Duesseldorf (intervention) and Wuppertal (control)

Supplementary Material 3. List of 65 baseline covariates in the PS model

Supplementary Material 4. TIDieR checklist

Supplementary Material 5. Comparison of socio-demographic characteristics of residents of the intervention (Duesseldorf) and control (Wuppertal) cities based on publicly available data and data from the study

## Data Availability

The datasets generated and/or analysed in this study are not publicly available due to the privacy of the participants, but can be obtained from the corresponding author upon reasonable request.

## Abbreviations

MVPA: moderate-to-vigorous physical activity
PA: Physical activity
WHO: World Health Organization
NAKO: National cohort health study
GEDA: Gesundheit in Deutschland aktuell
ALPHA: Instruments for assessing Levels of Physical Activity and Fitness
PS: Propensity score
GPAQ: Global Physical Activity Questionnaire
ICECAP: The ICEpop Capability measure for adults
CI: Confidence interval
SD: Standard deviation
SMD: Standardized mean difference
SOEP: Socio-economic panel
MDN: Median
Q1: First quartile
Q3: Third quartile
MET: Metabolic equivalents

## Footnotes

1 Komoot is a route planner, a navigation app, a tour directory and a social network for outdoor activities. It is aimed in particular at cyclists, hikers, mountain bikers, gravel bikers and racing cyclists.
