# Supplementary material for: Effects of the ‘10,000 Steps Duesseldorf' intervention promoting physical activity in community-dwelling adults: results of a nonrandomized controlled trial
Source: Int J Behav Nutr Phys Act. 2025 Dec 3;22:155. doi: 10.1186/s12966-025-01850-4 (PMC12690824; doi:10.1186/s12966-025-01850-4)
Supplement: Supplementary file 1 — Supplementary Material 1. Intervention components planned and implemented [file 12966_2025_1850_MOESM1_ESM.docx]

| Supplement 1: Overview of activities planned and implemented during the one-year intervention period. | |
| --- | --- |
|  |  |
| Planned | Implemented |
| Events organized by Duesseldorf’s administrative Office for Sports, city marathons, and events for health promotion organized by other entities | Informational event “Healthy employees - healthy company” (April 7-8^th^, 2022) by Chamber of Industry and Commerce |
|  | Volksgarten parkrun (August 27th and November 19^th^, 2022) |
|  | 10,000 steps Düsseldorf goes “sport in the park” (September 23^rd^, 2022) |
|  | Opening of mobility stations Kirchplatz (October 14, 2022), Bachplätzchen (December 16^th^, 2022) and Universität Mitte and Mensa (February 2^nd^, 2023) |
|  | Day of Movement at the German Diabetes Centre (November 12^th^, 2022) |
| Skyrun at the Duesseldorf television tower | Climbing the Duesseldorf television tower (June 8^th^, 2022) |
| City rallies with different local themes coming from arts, culture, and history | Night watchman tour (August 8^th^, 2022) |
|  | Büdchen route for the Büdchen day (August 13^th^, 2022) |
|  | Geocaches (published on September 12^th^, 2022) |
|  | Step competitions |
|  | o City-wide steps competition Düsseldorf (April 27^th^, 2022 and May 30^th^, 2022) |
|  | o Centers Plus - move more together! (October 17^th^, 2022) |
|  | o Make exercise a habit in your company! (November 7^th^, 2022) |
|  | o Ready for the island (January 9^th^, 2023) |
|  | o Fit for spring (March 20^th^, 2023) |
|  | o City district Battle (April 1^st^, 2023) |
| Family event in the local soccer stadium with representatives of the soccer team | Stadium tours in the Merkur Spiel-Arena (October 26^th^, 2022 and March 16^th^, 2023) |
