# Supplementary material for: Effects of the ‘10,000 Steps Duesseldorf' intervention promoting physical activity in community-dwelling adults: results of a nonrandomized controlled trial
Source: Int J Behav Nutr Phys Act. 2025 Dec 3;22:155. doi: 10.1186/s12966-025-01850-4 (PMC12690824; doi:10.1186/s12966-025-01850-4)
Supplement: Supplementary file 2 — Supplementary Material 2. City districts chosen for the study in Duesseldorf (intervention) and Wuppertal (control) [file 12966_2025_1850_MOESM2_ESM.docx]

**
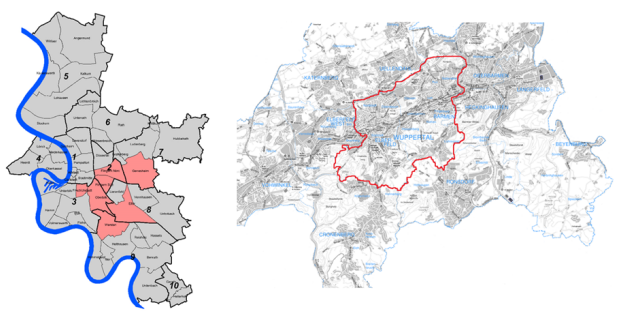
Supplement 2:** Intervention and control city district areas (left, source: Jugendamt Landeshauptstadt Düsseldorf [Youth Welfare Office, State capital Duesseldorf]; right, source: Offene Daten Wuppertal. *Matos Fialho et al.*^1^

*^1^Matos Fialho PM, Günther L, Schmitz E, et al. Effects of the Population-Based “10,000 Steps Duesseldorf” Intervention for Promoting Physical Activity in Community-Dwelling Adults: Protocol for a Nonrandomized Controlled Trial. JMIR Res Protoc. 2022;11(9):e39175. doi:10.2196/39175*
