# Supplementary material for: Effects of the ‘10,000 Steps Duesseldorf' intervention promoting physical activity in community-dwelling adults: results of a nonrandomized controlled trial
Source: Int J Behav Nutr Phys Act. 2025 Dec 3;22:155. doi: 10.1186/s12966-025-01850-4 (PMC12690824; doi:10.1186/s12966-025-01850-4)
Supplement: Supplementary file 3 — Supplementary Material 3. List of 65 baseline covariates in the PS model [file 12966_2025_1850_MOESM3_ESM.docx]

| Table 1. Characteristics of participants for all 65 variables included in propensity score analysis in the intervention (Duesseldorf, n=376) and control group (Wuppertal, n=251) before and after applying matching weights | | | | | | |
| --- | --- | --- | --- | --- | --- | --- |
| Demographics | **Duesseldorf baseline (*n*=376)** | **Wuppertal baseline (n=251)** | **z-difference** | **Duesseldorf baseline with matching weights (*n*=376)** | **Wuppertal baseline with matching weights (*n*=251)** | **z-difference** |
| Age (years) |  |  |  |  |  |  |
| Years (mean ± SD) | 55.3 ± 13.8 | 54.6 ± 13.4 | -0.67 | 55.6 ± 5.4 | 55.7 ± 6.2 | 0.02 |
| Gender (%) |  |  |  |  |  |  |
| Women | 62% | 57% | -1.28 | 61% | 61% | -0.05 |
| Level of education (%) |  |  |  |  |  |  |
| Primary school | 1% | 0% | 0.79 | 0% | 0% | 0.06 |
| Secondary school | 2% | 3% |  | 4% | 4% |  |
| Apprenticeship | 25% | 20% |  | 18% | 17% |  |
| A-levels | 18% | 21% |  | 24% | 25% |  |
| University degree | 50% | 54% |  | 53% | 52% |  |
| Promotion | 4% | 2% |  | 1% | 2% |  |
| Employment status (%) |  |  |  |  |  |  |
| Employed | 60% | 71% | -2.79 | 66% | 64% | 0.31 |
| Unemployed | 2% | 2% |  | 2% | 2% |  |
| Inactive persons | 38% | 27% |  | 32% | 34% |  |
| Occupation time (h/week) |  |  |  |  |  |  |
| Hours (mean ± SD) | 20.0 ± 18.7 | 23.7 ± 18.0 | 2.58 | 22.0 ± 7.4 | 21.9 ± 9.2 | -0.16 |
| Distance to occupation (km) |  |  |  |  |  |  |
| Km (mean ± SD) | 6.3 ± 11.3 | 8.5 ± 14.9 | 1.94 | 6.3 ± 4.5 | 6.8 ± 6.5 | -0.27 |
| Household income (Euro) |  |  |  |  |  |  |
| Euro (mean ± SD) | 2895 ± 1145 | 2660 ± 1518 | -1.07 | 2683 ± 612 | 2681 ± 773 | -0.01 |
| Migration background (%) |  |  |  |  |  |  |
| Yes | 32% | 36% | -0.83 | 35% | 38% | -0.43 |
| No | 66% | 63% |  | 63% | 60% |  |
| Unknown | 2% | 1% |  | 2% | 2% |  |
| Marital status (%) |  |  |  |  |  |  |
| Not married | 24% | 24% | 0.99 | 24% | 25% | 0.02 |
| Married and living together | 60% | 54% |  | 56% | 54% |  |
| Married but not living together | 2% | 3% |  | 3% | 3% |  |
| Divorced | 9% | 16% |  | 14% | 15% |  |
| Widowed | 4% | 3% |  | 3% | 3% |  |
| Unknown | 1% | 0% |  | 0% | 0% |  |
| Partnership (%) |  |  |  |  |  |  |
| No partner | 19% | 25% | 0.18 | 26% | 27% | -0.50 |
| Partner and living together | 75% | 62% |  | 63% | 64% |  |
| Partner but not living together | 6% | 13% |  | 11% | 9% |  |
| Type of housing |  |  |  |  |  |  |
| Rented apartment | 53% | 55% | -1.18 | 64% | 64% | 0.09 |
| Own apartment | 16% | 21% |  | 17% | 14% |  |
| Own house | 29% | 21% |  | 18% | 20% |  |
| Rented house | 2% | 2% |  | 1% | 2% |  |
| Assisted living | 0% | 0% |  | 0% | 0% |  |
| Retirement home | 0% | 0% |  | 0% | 0% |  |
| Unknown | 0% | 1% |  | 0% | 0% |  |
| Changes in work time due to COVID-19 (%) |  |  |  |  |  |  |
| Less work time | 8% | 14% | -0.46 | 12% | 10% | 0.00 |
| No change in work time | 80% | 71% |  | 76% | 79% |  |
| More work time | 12% | 15% |  | 12% | 11% |  |
| Changes in work due to COVID-19 (%) |  |  |  |  |  |  |
| New work | 3% | 4% | 0.92 | 4% | 4% | -0.07 |
| Loss of work | 2% | 2% | -0.69 | 4% | 3% | -0.17 |
| Change to home office | 35% | 36% | 0.43 | 33% | 34% | 0.06 |
| Short-time work allowance | 7% | 9% | 0.77 | 9% | 9% | -0.12 |
| Self-reported environmental factors |  |  |  |  |  |  |
| Density score (range: 63-315) | 161 ± 61 | 149 ± 58 | -2.59 | 152 ± 23 | 153 ± 30 | 0.09 |
| Distance score (range: 8-40) | 15.3 ± 4.7 | 16.7 ± 5.0 | 3.32 | 15.2 ± 1.9 | 15.4 ± 2.3 | 0.19 |
| Availability infrastructure (range: 4-16) | 11.6 ± 2.5 | 10.9 ± 2.7 | -3.65 | 11.4 ± 0.9 | 11.3 ± 1.2 | -0.32 |
| Total safety (range: 6-24) | 19.0 ± 3.6 | 18.9 ± 3.0 | -0.82 | 19.0 ± 1.2 | 19.1 ± 1.3 | -0.03 |
| Pleasure (range: 4-16) | 13.0 ± 2.2 | 12.6 ± 2.4 | -1.84 | 12.9 ± 0.9 | 13.0 ± 1.0 | 0.34 |
| Network (range: 4-16) | 12.8 ± 2.3 | 12.1 ± 2.2 | -4.02 | 12.5 ± 0.9 | 12.5 ± 0.9 | -0.40 |
| Home (range: 0-6) | 2.9 ± 1.0 | 2.8 ± 1.1 | -1.60 | 2.9 ± 0.4 | 2.8 ± 0.6 | 0.00 |
| Work/Study (range: 0-10) | 3.3 ± 1.6 | 3.4 ± 1.7 | 1.27 | 3.3 ± 0.6 | 3.2 ± 0.8 | -0.17 |
| Hilly roads (range: 1-4) | 1.4 ± 0.8 | 3.3 ± 1.1 | 17.43 | 2.3 ± 0.4 | 2.3 ± 0.6 | -0.06 |
| Hilly environment (range: 1-4) | 1.2 ± 0.6 | 2.3 ± 1.1 | 13.22 | 1.7 ± 0.3 | 1.8 ± 0.4 | 0.03 |
| Self-reported PA (MET-min/week) |  |  |  |  |  |  |
| Work-related intensive PA | 635 ± 1983 | 800 ± 2561 | 0.20 | 716 ± 765 | 747 ± 1071 | -0.11 |
| Work-related moderate PA | 318 ± 991 | 400 ± 1280 | 0.20 | 358 ± 382 | 373 ± 535 | -0.11 |
| Locomotion by foot or bicycle | 856 ± 973 | 822 ± 1012 | -1.46 | 837 ± 356 | 868 ± 473 | 0.22 |
| Leisure time intensive PA | 643 ± 1004 | 935 ± 1587 | 1.83 | 695 ± 405 | 733 ± 601 | -0.10 |
| Leisure time moderate PA | 701 ± 1246 | 824 ± 1194 | 2.09 | 803 ± 612 | 834 ± 510 | 1.02 |
| Sedentary behaviour (min/day) |  |  |  |  |  |  |
| Minutes per day (mean ± SD) | 409 ± 204 | 419 ± 198 | 0.72 | 405 ± 82 | 409 ± 85 | 0.41 |
| Pedometer-determined PA (steps/day) |  |  |  |  |  |  |
| Steps per day (mean ± SD) | 7989 ± 3358 | 8022 ± 3640 | -0.28 | 8129 ± 1455 | 8163 ± 1842 | -0.13 |
| Changes in PA at work due to COVID-19 (%) |  |  |  |  |  |  |
| Much less than before | 12% | 12% | -0.72 | 16% | 14% | 0.38 |
| Slightly less than before | 8% | 13% |  | 10% | 11% |  |
| Remained the same | 60% | 55% |  | 52% | 52% |  |
| Slightly more than before | 4% | 6% |  | 5% | 5% |  |
| Much more than before | 3% | 3% |  | 3% | 2% |  |
| No work | 13% | 11% |  | 13% | 16% |  |
| Changes in PA in household due to COVID-19 (%) |  |  |  |  |  |  |
| Much less than before | 1% | 2% | -1.40 | 0% | 1% | -0.04 |
| Slightly less than before | 1% | 2% |  | 2% | 1% |  |
| Remained the same | 72% | 74% |  | 69% | 70% |  |
| Slightly more than before | 19% | 17% |  | 19% | 18% |  |
| Much more than before | 7% | 5% |  | 10% | 10% |  |
| Changes in PA in leisure due to COVID-19 (%) |  |  |  |  |  |  |
| Much less than before | 9% | 6% | 0.45 | 9% | 9% | 0.13 |
| Slightly less than before | 9% | 14% |  | 14% | 13% |  |
| Remained the same | 41% | 37% |  | 42% | 43% |  |
| Slightly more than before | 27% | 26% |  | 20% | 20% |  |
| Much more than before | 14% | 17% |  | 15% | 15% |  |
| Changes in PA in sports due to COVID-19 (%) |  |  |  |  |  |  |
| Much less than before | 24% | 19% | 0.64 | 23% | 24% | -0.07 |
| Slightly less than before | 13% | 17% |  | 13% | 12% |  |
| Remained the same | 47% | 45% |  | 48% | 47% |  |
| Slightly more than before | 10% | 12% |  | 9% | 9% |  |
| Much more than before | 6% | 7% |  | 7% | 8% |  |
| Changes in PA in locomotion due to COVID-19 (%) |  |  |  |  |  |  |
| Much less than before | 6% | 6% | 1.25 | 7% | 6% | 0.14 |
| Slightly less than before | 11% | 10% |  | 13% | 13% |  |
| Remained the same | 59% | 54% |  | 54% | 54% |  |
| Slightly more than before | 15% | 21% |  | 19% | 19% |  |
| Much more than before | 9% | 9% |  | 7% | 8% |  |
| Changes in PA in sitting due to COVID-19 (%) |  |  |  |  |  |  |
| Much less than before | 1% | 2% | -0.54 | 0% | 0% | -0.16 |
| Slightly less than before | 3% | 5% |  | 3% | 3% |  |
| Remained the same | 57% | 54% |  | 57% | 58% |  |
| Slightly more than before | 20% | 25% |  | 18% | 20% |  |
| Much more than before | 19% | 14% |  | 22% | 19% |  |
| Self-control score |  |  |  |  |  |  |
| Self-control score | 3.1 ± 0.7 | 3.2 ± 0.7 | 0.86 | 3.2 ± 0.3 | 3.2 ± 0.3 | 0.06 |
| Pre-existing comorbidities (%) |  |  |  |  |  |  |
| Asthma | 10% | 12% | 0.68 | 18% | 16% | -0.49 |
| COPD | 7% | 6% | -0.67 | 10% | 10% | -0.14 |
| Cardiovascular diseases | 5% | 3% | -0.98 | 3% | 3% | 0.07 |
| Arthrosis | 24% | 21% | -0.75 | 23% | 22% | -0.30 |
| Back pain | 37% | 39% | 0.42 | 37% | 39% | 0.20 |
| Neck pain | 28% | 29% | 0.13 | 31% | 29% | -0.25 |
| Diabetes | 6% | 7% | 0.66 | 8% | 10% | 0.46 |
| Allergies | 37% | 33% | -1.03 | 37% | 35% | -0.30 |
| Liver cirrhosis | 1% | 0.5% | -0.62 | 2% | 2% | -0.05 |
| Urinary incontinence | 6% | 5% | -0.62 | 5% | 5% | -0.10 |
| Chronic kidney disease | 3% | 2% | -0.88 | 2% | 2% | -0.15 |
| Depression | 11% | 10% | -0.58 | 9% | 9% | -0.20 |
| Hypertension | 36% | 36% | -0.15 | 40% | 42% | 0.29 |
| Hyperlipidaemia | 34% | 43% | 2.30 | 31% | 32% | 0.24 |
| Days of sick leave (days) |  |  |  |  |  |  |
| Days (mean ± SD) | 5.5 ± 24.5 | 4.6 ± 16.5 | -0.01 | 4.1 ± 4.4 | 4.6 ± 7.3 | 0.41 |
| Hospital visit in the last 6 months (%) |  |  |  |  |  |  |
| Yes | 6% | 8% | 0.58 | 6% | 6% | 0.08 |
| Intake of fruit and vegetables (%) |  |  |  |  |  |  |
| Daily | 77% | 77% | 0.31 | 78% | 77% | 0.02 |
| 4-6 times / week | 16% | 11% |  | 13% | 14% |  |
| 1-3 times / week | 6% | 11% |  | 9% | 9% |  |
| Less than once / week | 0.5% | 0.5% |  | 0% | 0% |  |
| Never | 0% | 0.5% |  | 0% | 0% |  |
| Alcohol intake |  |  |  |  |  |  |
| Alcohol audit score (range: 0-7) | 2.4 ± 1.5 | 2.1 ± 1.5 | -3.10 | 2.2 ± 0.6 | 2.2 ± 0.7 | -0.23 |
| Smoking (%) |  |  |  |  |  |  |
| Daily | 10% | 15% | -1.72 | 12% | 13% | 0.12 |
| Sometimes | 4% | 3% |  | 4% | 3% |  |
| Former | 36% | 38% |  | 34% | 34% |  |
| Never | 50% | 44% |  | 50% | 50% |  |
| Body mass index (kg/m²) |  |  |  |  |  |  |
| Kg/m² (mean ± SD) | 25.8 ± 4.9 | 25.9 ± 4.6 | 0.66 | 26.1 ± 1.7 | 26.1 ± 2.3 | -0.21 |
| Self-reported state of health (%) |  |  |  |  |  |  |
| Excellent | 5% | 5% | 0.38 | 4% | 4% | 0.26 |
| Very good | 27% | 27% |  | 25% | 26% |  |
| Good | 53% | 51% |  | 53% | 51% |  |
| Poor | 12% | 16% |  | 16% | 17% |  |
| Weak | 3% | 1% |  | 2% | 2% |  |
| Quality of life |  |  |  |  |  |  |
| EQ-5D-5L (range: -0.6-1) | 0.91 ± 0.14 | 0.90 ± 0.13 | -1.56 | 0.91 ± 0.04 | 0.90 ± 0.07 | 0.42 |
| Capability |  |  |  |  |  |  |
| ICECAP-A (range: 0-1) | 0.87 ± 0.10 | 0.87 ± 0.11 | 0.50 | 0.87 ± 0.04 | 0.87 ± 0.06 | -0.02 |
| Social support |  |  |  |  |  |  |
| Family support (range: 1-4) | 1.8 ± 0.6 | 1.8 ± 0.7 | -0.39 | 1.8 ± 0.2 | 1.8 ± 0.3 | -0.99 |
| Friends support (range: 1-4) | 1.6 ± 0.6 | 1.6 ± 0.7 | 0.27 | 1.6 ± 0.2 | 1.6 ± 0.3 | -0.61 |
