# Supplementary material for: Effects of the ‘10,000 Steps Duesseldorf' intervention promoting physical activity in community-dwelling adults: results of a nonrandomized controlled trial
Source: Int J Behav Nutr Phys Act. 2025 Dec 3;22:155. doi: 10.1186/s12966-025-01850-4 (PMC12690824; doi:10.1186/s12966-025-01850-4)
Supplement: Supplementary file 5 — Supplementary Material 5. Comparison of socio-demographic characteristics of residents of the intervention (Duesseldorf) and control (Wuppertal) cities based on publicly available data and data from the study [file 12966_2025_1850_MOESM5_ESM.pdf]

Supplement 5: Comparison of socio-demographic characteristics of residents of the intervention (Duesseldorf) and control (Wuppertal) cities based on publicly available data and data from the study.

Indicator	Duesseldorf – publicly available data (2022)*	Duesseldorf – study data	Wuppertal – publicly available data (2022/2021)**	Wuppertal – study data
**Total population**	653,253	376	365,958	251
**Gender (%)**				
**Female**	51.4	61.7	50.5	56.6
**Male**	48.6	38.3	49.4	43.4
**Age group (%), years**				
**0-17**	16.0	-	17.4	-
**18-29**	14.7	1.6	8.0	4.8
**30-49**	30.0	37.2	26.3	29.9
**50-64**	20.6	24.7	27.7	39.8
**65+**	18.7	36.4	20.5	25.5
**Migration background (%)**	25.1	32.4	22.1	35.5

* https://opendata.duesseldorf.de/

** https://www.offenedaten-wuppertal.de/
